# PDX regulates inflammatory cell infiltration via resident macrophage in LPS‐induced lung injury

**DOI:** 10.1111/jcmm.15679

**Published:** 2020-07-31

**Authors:** Yang Ye, Hua‐Wei Zhang, Hong‐Xia Mei, Hao‐Ran Xu, Shu‐Yang Xiang, Qian Yang, Sheng‐Xing Zheng, Fang Gao Smith, Sheng‐Wei Jin, Qian Wang

**Affiliations:** ^1^ Department of Anesthesia and Critical Care the Second Affiliated Hospital and Yuying Children's Hospital of Wenzhou Medical University Zhejiang China; ^2^ Institute of Inflammation and Aging College of Medical and Dental Sciences University of Birmingham Birmingham UK

**Keywords:** acute respiratory distress syndrome, neutrophil, protectin DX, recruited macrophage, resident macrophage

## Abstract

Inflammatory cell infiltration contributes to the pathogenesis of acute respiratory distress syndrome (ARDS). Protectin DX (PDX), an endogenous lipid mediator, shows anti‐inflammatory and proresolution bioactions. In vivo, the mice were intraperitoneally injected with PDX (0.1 µg/mouse) after intratracheal (1 mg/kg) or intraperitoneal (10 mg/kg) LPS administration. Flow cytometry was used to measure inflammatory cell numbers. Clodronate liposomes were used to deplete resident macrophages. RT‐PCR, and ELISA was used to measure MIP‐2, MCP‐1, TNF‐α and MMP9 levels. In vitro, sorted neutrophils, resident and recruited macrophages (1 × 10^6^) were cultured with 1 μg/mL LPS and/or 100 nmol/L PDX to assess the chemokine receptor expression. PDX attenuated LPS‐induced lung injury via inhibiting recruited macrophage and neutrophil recruitment through repressing resident macrophage MCP‐1, MIP‐2 expression and release, respectively. Finally, PDX inhibition of neutrophil infiltration and transmembrane was associated with TNF‐α/MIP‐2/MMP9 signalling pathway. These data suggest that PDX attenuates LPS‐stimulated lung injury via reduction of the inflammatory cell recruitment mediated via resident macrophages.

## 1 INTRODUCTION

Acute respiratory distress syndrome (ARDS) is a foetal disease,^1^ the hallmarks of which are massive pro‐inflammatory cytokine secretion and pro‐inflammatory cell infiltration.^2^, ^3^, ^4^ Emerging evidence has revealed that macrophages, containing resident macrophages and circulating monocyte‐derived macrophages, are central in the pathogenesis of ARDS.^5^, ^6^

Resident macrophages reside in the alveolar space and perform tissue‐specific, homeostatic functions.^7^ Growing evidence indicating that resident macrophage depletion protects against lung injury.^1^ However, recruited macrophages generate cytokines that are connected with glycolytic and arginine metabolism.^8^ A recent study showed that in a liposaccharide (LPS)‐induced ARDS mouse model, depletion of circulating monocytes attenuated lung infiltration by neutrophils and the severity of ARDSI.^9^, ^10^

Many cytokines, such as IL‐6, TNF‐α and matrix metalloproteinase 9 (MMP9), exhibit elevated expression in ARDS. Moreover, many chemokines are also involved in ARDS, such as macrophage inflammatory protein 2 (MIP‐2), monocyte chemoattractant protein‐1 (MCP‐1).^9^, ^11^, ^12^ MCP‐1 is a widely expressed chemoattractant of monocytes and macrophages. In the lungs, MCP‐1 enhances monocyte trafficking and alveolar macrophage pool expansion, and MIP‐2 promotes neutrophils recruitment through binding to CCR2 and CXCR2, respectively.^12^, ^13^, ^14^, ^15^

Protectin DX (10S,17S‐dihydroxydocosa‐4Z,7Z,11E,13Z,15E,19Z‐hexaenoic acid) (PDX), an endogenous lipid mediator, exerts potent anti‐inflammatory and proresolution bioactions, including inhibiting neutrophil infiltration in murine peritonitis.^16^, ^17^ Previous study showed that PDX reduced LPS‐induced secretion of pro‐inflammatory cytokines, such as TNF‐α and MCP‐1.^18^

We previously reported that PDX promoted alveolar fluid clearance and alleviated lung injury.^16^ We also found that PDX attenuated bleomycin‐induced lung fibrosis and dysfunction in mice.^19^

In the present study, we hypothesize that PDX attenuates LPS‐induced lung injury via inhibiting inflammatory cells recruitment. The secondary hypothesis is that PDX reduces recruited macrophage and neutrophil recruitment via repressing resident macrophage MCP‐1, MIP‐2 expression and release, respectively. Finally, we have been suggested that PDX inhibits neutrophil infiltration and transmembrane was associated with TNF‐α/MIP‐2/MMP9 signalling pathway.

## 2 MATERIALS AND METHODS

### 2.1 Materials

PDX was from Cayman Chemical Company (Ann Arbor, MI, USA). LPS (*Escherichia coli* serotype 055:B5) was from Sigma (St. Louis, MO, USA). TNF‐α, MIP‐2, MCP‐1 and MMP9 ELISA kits were from R&D Systems (Minneapolis, MN, USA). CXCR2 inhibitor, CCR2 inhibitor, TNF‐α inhibitor and MMP9 inhibitor were from MedChem Express (Monmouth Junction, NJ, USA). Anti‐MIP‐2 FITC‐conjugated, anti‐MCP‐1 FITC‐conjugated, anti‐Ly6c FITC‐conjugated, anti‐Ly6g FITC‐conjugated, anti‐F4/80 PE‐Cyanine7‐conjugated, anti‐CD11c PerCP‐Cyanine5.5‐conjugated and anti‐CD11b APC‐conjugated antibodies were from Invitrogen (Carlsbad, CA, USA).

### 2.2 Animal preparation

C57BL/6 mice (20‐25 g) were obtained from Slac Laboratory Animal (Shanghai, China). Mice were caged with free access to food and fresh water in a temperature‐controlled room on a standard day‐night cycle. The use of animals in the present study was approved by Animal Studies Ethics Committee of the Second Affiliated Hospital of Wenzhou Medical University.

For the experiment groups, mice received PDX (0.1 µg/mouse, intraperitoneal, ip) 10 minutes after atomization inhalation of LPS (ih, 1 mg/kg) or intraperitoneal injection of LPS (ip, 1 mg/kg). For the inhibitor groups, mice received the CXCR2 inhibitor (CXCR2i, 2 mg/kg), CCR2 inhibitor (CCR2i, 30 mg/kg), TNFR inhibitor (TNFRi, 15 mg/kg) or MMP9 inhibitor (MMP9i, 10 mg/kg) with or without PDX after LPS interruption. 24 hours later, the bronchoalveolar lavage fluid (BALF) and lung tissue samples were harvested.

### 2.3 Pathological studies

Lung lobe was collected and fixed in 4% paraformaldehyde for 24 hours, then embedded in paraffin and stained with haematoxylin and eosin (H&E) for light microscopy. Alveolar congestion, haemorrhage, neutrophil infiltration or aggregation, alveolar wall thickness/hyaline membrane formation were used to assay lung injury.^20^ No injury = 0; slight injury = 1 (25%); moderate injury = 2 (50%); severe injury = 3 (75%); and very severe injury = 4 (almost 100%).

### 2.4 Flow cytometry

Freshly collected and isolated BALF cells were incubated with anti‐F4/80 PE‐Cyanine7‐conjugated, anti‐Ly6c FITC‐conjugated, anti‐Ly6g FITC‐conjugated, anti‐CD11b APC‐conjugated and anti‐CD11c PerCP‐Cyanine5.5‐conjugated antibodies for 30 minutes. Then cells were cultured with fluorescence‐activated cell sorting (FACS) lysis solution for another 10 minutes. After 5 minutes 400 *g* centrifuge, cells were analysed by CytExpert 2.0 (Beckman Coulter).

Macrophage was identified by high expression of F4/80. Resident macrophage was defined by high expression of CD11b, and recruited macrophage was defined by high expression of CD11c, Ly6c. Neutrophil was identified by high expression of Ly6c and Ly6g.

### 2.5 Resident macrophages depletion

To deplete resident macrophages, clodronate liposome was given intratracheal in a volume of 50 µl (5 mg/mL) 72 hours before LPS challenge. PBS liposome was used as control. Next, mice were stimulated with LPS (1 mg/kg) with or without PDX (0.1 µg/mouse) for 24 hours. Then BALF was harvested.

### 2.6 Quantitative real‐time RT‐PCR

Total RNA from lung tissues was isolated using TRIzol reagent (Invitrogen, Carlsbad, CA), according to the manufacturer's protocol. The cDNA was synthesized using a reverse transcription Kit. Gene expression was detected using SYBR green super‐mix PCR kit. Then MIP‐2, MCP‐1 and TNF‐α mRNA level were measured.

The primer pairs for each gene were as follows: MIP‐2:5′‐CCACTCACCTGCTGCTACTCATTC‐3′ and reverse 5′‐CTGCTGCTGGTGATCCTCTTGTAG‐3′; MCP‐1:5‐CCACTCACCTGCTGCTACTCATTC‐3, 5‐CTGCTGCTGGTGATCCTCTTGTAG‐3; and TNF‐α: 5‐GCGACGTGGAACTGGCAGAAG‐3, 5‐GCCACAAGCAGGAATGAGAAGAGG‐3.

Next, Neutrophils, resident and recruited macrophages were sorted and stimulated with 1 μg/mL LPS and/or 100 nmol/L PDX for 24 hours, then CXCR2, CCR2 and TNFR expression were assayed by real‐time PCR.

### 2.7 ELISA

MIP‐2, MCP‐1, TNF‐α and MMP‐9 concentrations in lung tissue homogenates and BALF were assessed using ELISA kits, according to the manufacturer's protocol.

### 2.8 Statistical analysis

Data are presented as the mean ± SEM. All data were analysed by one‐way ANOVA, followed by Tukey test for post hoc comparison. Significance was considered at the *P* < .05. Statistical analyses were performed using Prism 6.0 software (GraphPad Software, San Diego, CA, USA).

## 3 RESULTS

### 3.1 PDX protected lung tissue from LPS‐induced lung injury

**FIGURE 1:**
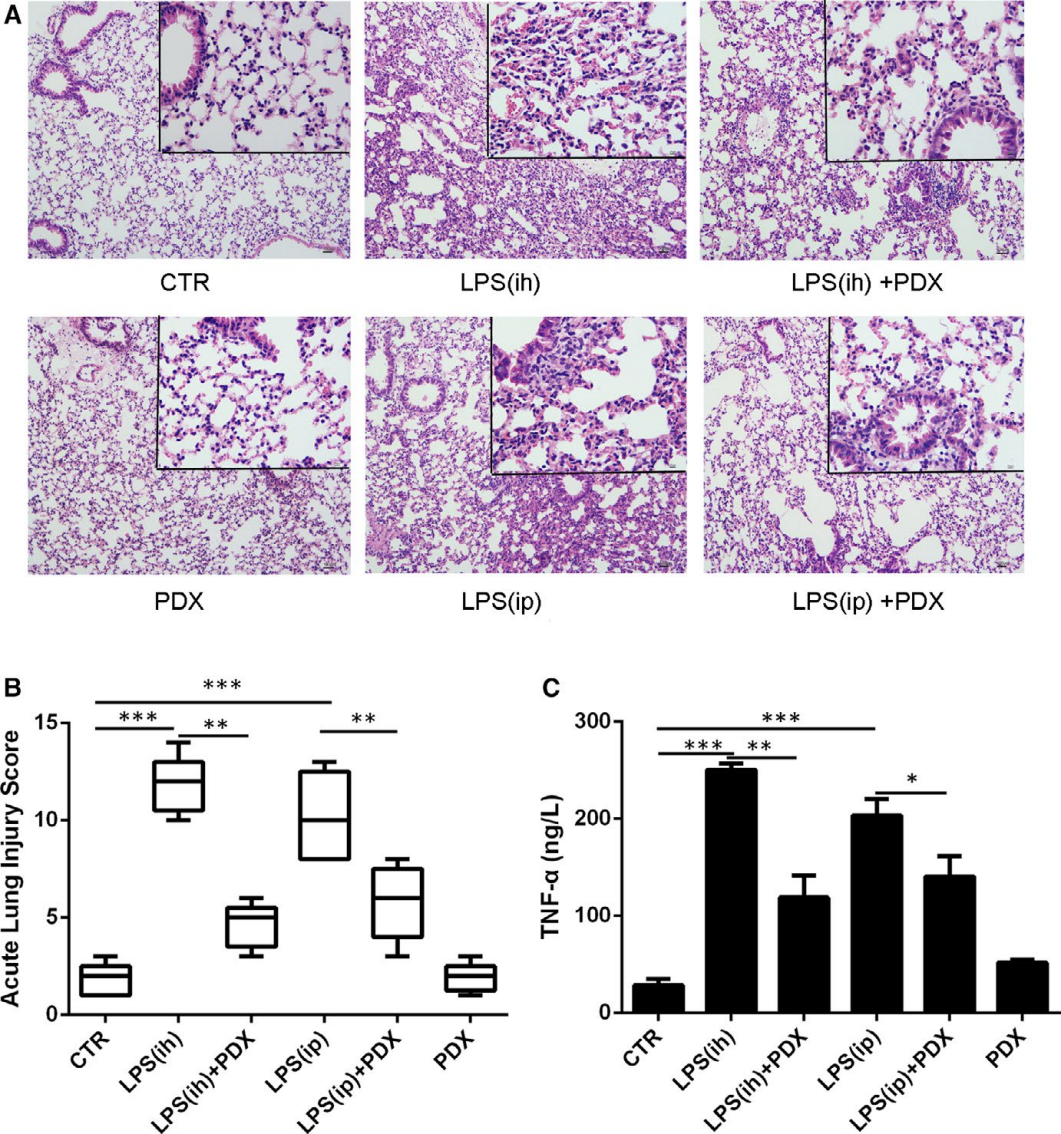
PDX attenuated LPS‐induced lung tissue damage. Mice received LPS by intratracheal atomization (ih) (1 mg/kg) or intraperitoneal injection (ip) (10 mg/kg) and then received PDX (0.1 µg/mouse) by intraperitoneal injection. Lung histological changes were assessed 24 h later by haematoxylin and eosin staining (A) and acute lung injury scoring (B). Aerosol inhalation and intraperitoneal injection of LPS both significantly increased the TNF‐α concentration in lung tissue homogenates, and this effect was markedly attenuated by PDX treatment. Data are presented as the mean ± SEM. n = 6‐8. *P < .05, **P < .01, ***P < .001

As shown in Figure 1A, the lung tissues were seriously injured in both LPS (ih) and LPS (ip) groups. Treatment with PDX alleviated LPS‐induced lung injury. Acute lung injury scores were in line with pathophysiological changes (Figure 1B). In addition, the lung tissue homogenate TNF‐α level was significantly higher in both the LPS (ih) and LPS (ip) groups than in the CTR group and was lower in the PDX treatment group (Figure 1C). But there were no significant differences between the CTR group and PDX group (*P* > .05).

### 3.2 PDX reduced inflammatory cell accumulation in LPS‐induced lung injury

**FIGURE 2:**
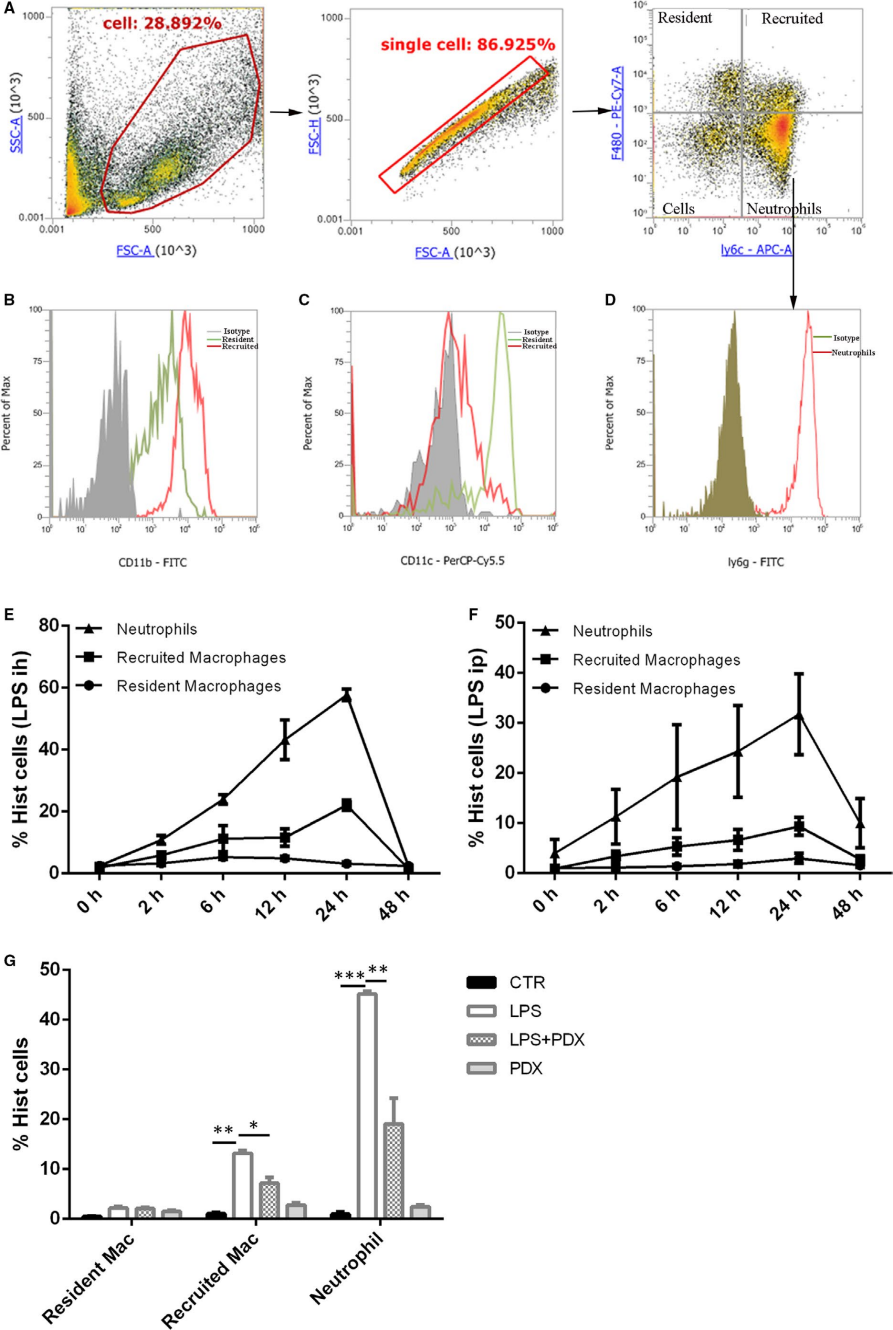
PDX reduced recruited macrophage and neutrophil infiltration in LPS‐induced lung injury in vivo. F4/80^‐^Ly6c^+^Ly6g^+^ neutrophils, F4/80^+^Ly6C^‐^CD11c^hi^CD11b^int^ resident macrophages and F4/80^+^Ly6c^+^CD11c^lo^CD11b^hi^ recruited macrophages in the BALF were separated by flow cytometry (A). The numbers of neutrophils, resident macrophages and recruited macrophages in the BALF were determined by flow cytometry after LPS inhalation (1 mg/kg) (B) or intraperitoneal injection (10 mg/kg) (C). Next, PDX (0.1 µg/mouse) was administered to mice 10 min after LPS (1 mg/kg) inhalation; 24 h later, the three cell types were counted by flow cytometry (D). Data are presented as the mean ± SEM. n = 6‐8. *P < .05, **P < .01, ***P < .001

F4/80^‐^Ly6c^+^Ly6g^+^ neutrophil, F4/80^+^Ly6C^‐^CD11c^hi^CD11b^int^ resident macrophage and F4/80^+^Ly6c^+^CD11c^lo^CD11b^hi^ recruited macrophage (Figure 2A‐D) in the BALF were separated as previously described.^21^

In the LPS‐ (ih) (Figure 2E) or LPS (ip) (Figure 2F)‐induced lung injury model, neutrophils started infiltrating the lungs at 2 hours after LPS intervention, and the number of neutrophils peaked at 24 hours and then decreased until 48 hours. The number of recruited macrophages was increased at 6 hours, peaked at 24 hours and then gradually decreased until 48 hours, but the number of resident macrophages generally remained constant during the course of LPS‐induced inflammation. Inflammatory cell numbers were much more stable in the LPS (ih) group (Figure 2E) than in the LPS (ip) group (Figure 2F). Therefore, the LPS inhalation (ih)‐induced lung injury model was used in subsequent experiments.

The recruited macrophages and neutrophils were decreased at 24 hours in the LPS + PDX group compared with the LPS group (Figure 2G) (*P* < .05). But there was no significant difference between the CTR group and PDX group (Figure 2G) (*P* > .05).

### 3.3 PDX reduced inflammatory cell infiltration in LPS‐induced lung injury via resident macrophages

**FIGURE 3:**
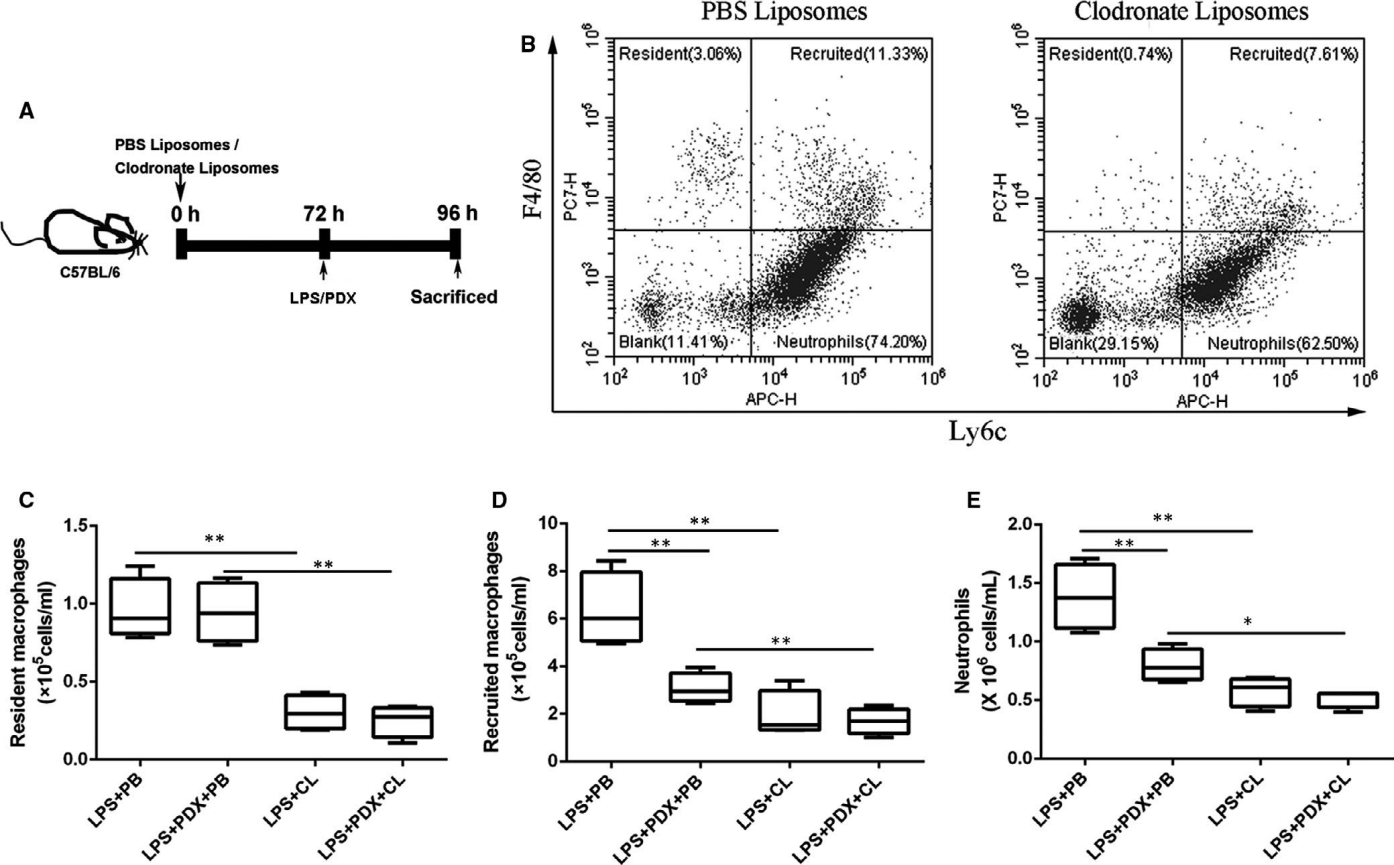
The inhibition of LPS‐induced inflammatory cell infiltration by PDX was dependent on resident macrophages. Resident macrophages were depleted in the lungs 72 h after intratracheal administration of 50 µl clodronate liposomes, and the administration of PDX (0.1 µg/mouse) occurred 10 min after LPS (1 mg/kg) stimulation (A). The numbers of resident macrophages (B, C), recruited macrophages (B, D) and neutrophils (B, E) in the BALF were measured by flow cytometry. CL = clodronate liposome, PB = PBS liposome. The data are presented as the mean ± SEM. n = 6‐8. *P < .05, **P < .01, ***P < .001

Clodronate liposome was used to eliminate resident macrophage (Figure 3A). As shown in Figure 3B, clodronate liposome depleted the vast majority of resident macrophages. Compared with LPS + PBS liposome group, the recruited macrophages and neutrophils were decreased in LPS + PDX+PBS liposome group (*P* < .05), but not resident macrophages (*P* > .05) (Figure 3C‐E), suggesting that PDX reduced inflammatory cells infiltration after LPS challenge, but had no effect on resident macrophage numbers. Compared with LPS + PBS liposome group, the resident macrophages, recruited macrophages and neutrophils were decreased in LPS + clodronate liposome group (*P* < .05), indicating that while clodronate liposome eliminates resident macrophages, recruited macrophage and neutrophils numbers also reduced (*P* < .05) (Figure 3C‐E). However, PDX had no effect on inflammatory cell numbers after clodronate liposome stimulation (Figure 3D,E), indicating that PDX reduced inflammatory cell infiltration in LPS‐induced lung injury via resident macrophages.

### 3.4 PDX reduced resident macrophage MIP‐2 and MCP‐1 production and release in LPS‐induced lung injury

**FIGURE 4:**
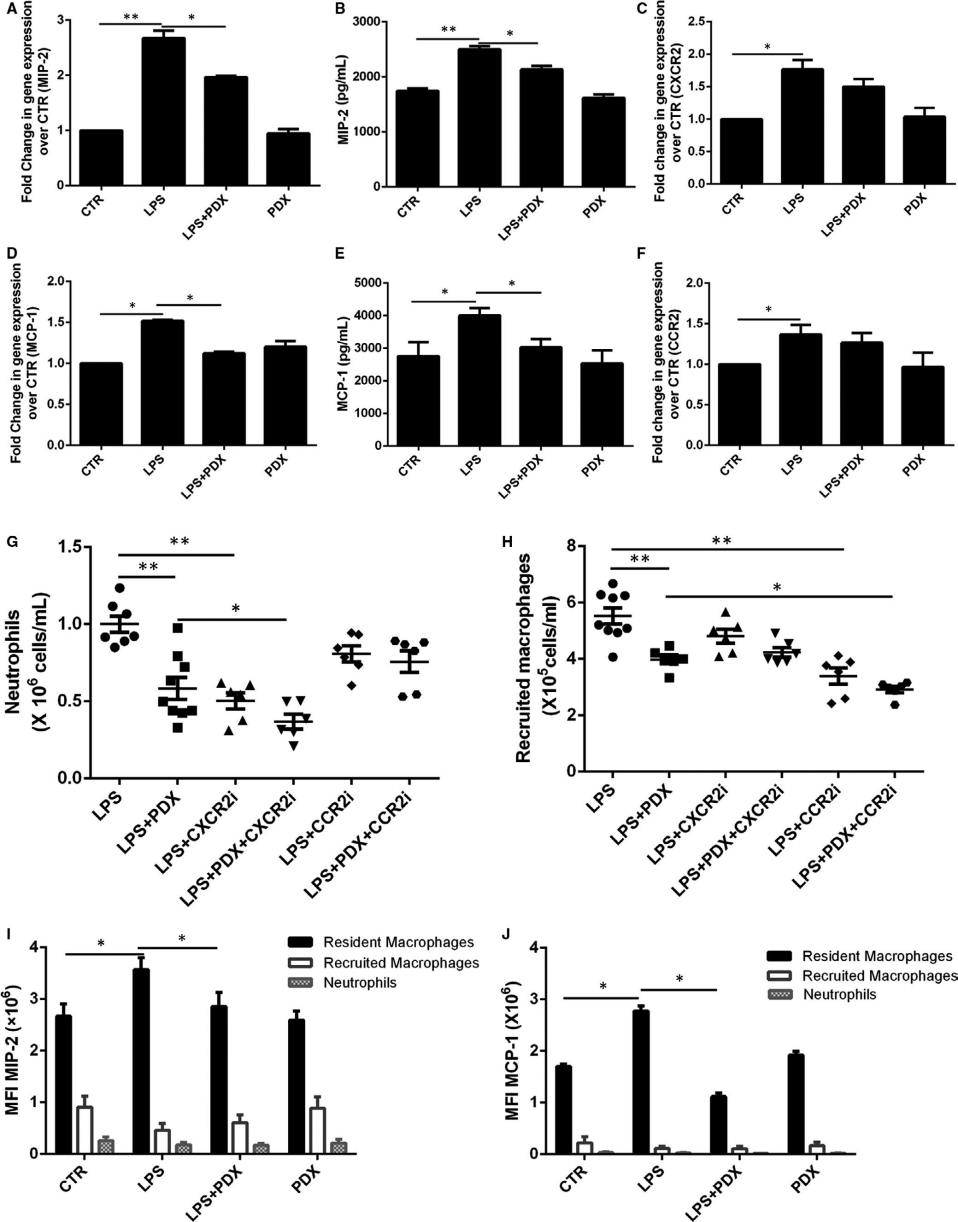
PDX down‐regulated LPS‐stimulated resident macrophage MIP‐2 and MCP‐1 expression and release to inhibit inflammatory cell infiltration. Mice received 1 mg/kg LPS by intratracheal atomization and then received PDX (0.1 µg/mouse) by intraperitoneal injection. MIP‐2 and MCP‐1 mRNA expression in lung tissue homogenates (A) and protein levels in the BALF (B) were measured 24 h later. Next, the sorted neutrophils (1 × 10^6^) and recruited macrophages (1 × 10^6^) were incubated with LPS (1 μg/mL) in the presence or absence of PDX (100 nmol/mL) for 24 h, respectively. CXCR2 mRNA expression on neutrophils and CCR2 mRNA level on recruited macrophages were measured by real‐time PCR (C,F). In addition, mice received a CXCR2 inhibitor (2 mg/kg) or CCR2 inhibitor (30 mg/kg) in the presence or absence of PDX via intraperitoneal injection 10 min after LPS administration. The number of neutrophils (G) and recruited macrophages (H) in the BALF was evaluated by flow cytometry. In addition, the mean fluorescence intensity (MFI) of MIP‐2 (I) and MCP‐1 (J) was assessed by flow cytometry. MIP‐2 and MCP‐1 were mainly expressed on resident macrophages, but this expression was strongly down‐regulated by PDX (I, J). CXCR2i = CXCR2 inhibitor, CCR2i = CCR2 inhibitor. Data are presented as the mean ± SEM. n = 6‐8. *P < .05, **P < .01

LPS application not only increased MIP‐2 and MCP‐1 mRNA expression in tissues, but also up‐regulated MIP‐2 and MCP‐1 level in the BALF compared with CTR treatment (*P* < .05). Treatment with PDX observably weakened the MIP‐2 and MCP‐1 concentration in tissues and BALF compared with LPS group (*P* < .05) (Figure 4A,B,D,E). However, there was no difference in the CXCR2 expression on neutrophils and CCR2 expression on recruited macrophages among these groups (*P* > .05) (Figure 4C,F).

Next, CXCR2i (MIP‐2 receptor inhibitor) and CCR2i (MCP‐1 receptor inhibitor) were administered via intraperitoneal injection. As shown in Figure 4G,H, with or without PDX, treatment with CXCR2i and CCR2i reduced the number of neutrophils and recruited macrophages, respectively, indicating that the basic function of PDX was disappeared after using CXCR2 and CCR2 inhibitors.

In addition, Figure 4I,J showed that MIP‐2 and MCP‐1 were mostly presented on resident macrophages, and LPS stimulation increased the MIP‐2 and MCP‐1 mean fluorescence intensity (MFI). Furthermore, the up‐regulation of MIP‐2 and MCP‐1 expression induced by LPS was reduced by PDX.

### 3.5 PDX reduced neutrophil recruitment via recruited macrophage TNF‐α/MIP‐2 signalling pathway

**FIGURE 5:**
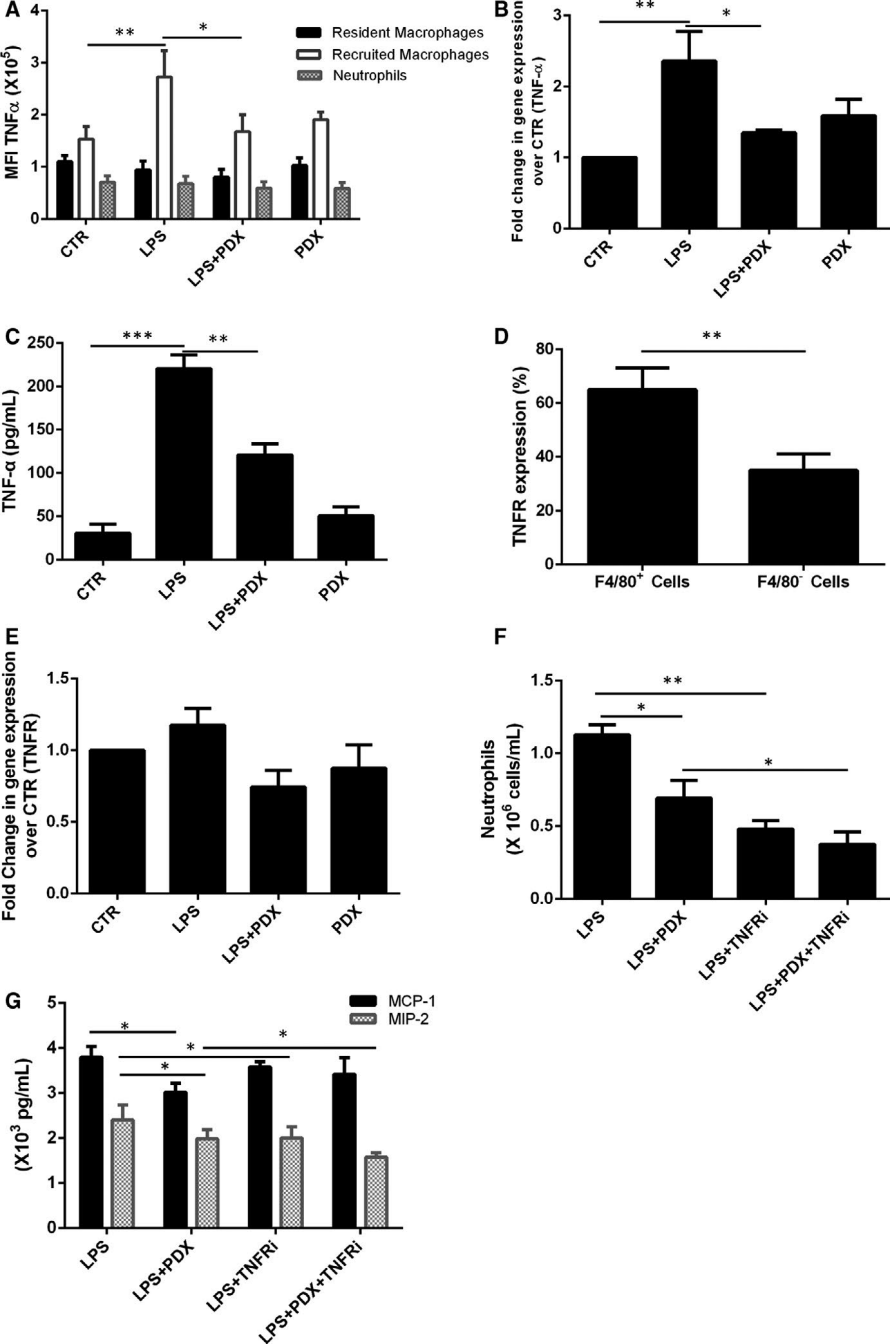
PDX inhibited neutrophil infiltration via recruited macrophage TNF‐α/MIP‐2 signalling pathway. Mice received 1 mg/kg LPS by intratracheal atomization and then received PDX (0.1 µg/mouse) by intraperitoneal injection. Lung homogenates were collected and processed into single‐cell suspensions 24 h later. The mean fluorescence intensity (MFI) of TNF‐α was assessed by flow cytometry (A). TNF‐α was mainly expressed on resident macrophages, but this expression was strongly down‐regulated by PDX (B). TNF‐α mRNA expression in lung tissue homogenates (B) and TNF‐α protein levels in the BALF (C) were measured 24 h later. Then, TNF‐α expression in different kinds of cells was assessed by flow cytometry (D). Next, the sorted resident macrophages (1 × 10^6^) were incubated with LPS (1 μg/mL) in the presence or absence of PDX (100 nmol/mL) for 24 h. TNF‐α mRNA expression on resident macrophages was measured by real‐time PCR (E). In addition, mice received a TNFR inhibitor (15 mg/kg) in the presence or absence of PDX via intraperitoneal injection 10 min after LPS administration. The number of neutrophils (F) in the BALF was evaluated by flow cytometry, and the MIP‐2, MCP‐1 level was measured by ELISA (G). TNFRi = TNFR inhibitor. Data are presented as the mean ± SEM. n = 6‐8. *P < .05, **P < .01

As shown in Figure 5A, TNF‐α was mostly presented on recruited macrophages, and treatment with PDX suppressed the TNF‐α MFI compared with LPS group (*P* < .05). Up‐regulation of lung tissue TNF‐α mRNA and BALF TNF‐α concentration in the LPS group could be eliminated by PDX treatment (Figure 5B,C). Figure 5D showed that TNFR was mainly presented on macrophages, but there was no significant difference among these groups (*P* > .05) (Figure 5E).

In addition, TNFRi (a TNF‐α receptor inhibitor) was administered via intraperitoneal injection. With or without PDX, treatment with TNFRi reduced neutrophil numbers and MIP‐2 level, but not MCP‐1, indicating that the basic function of PDX was disappears after using TNFR inhibitors (Figure 5F,G).

### 3.6 PDX inhibited neutrophil transmigration into the alveolar space in connection with TNF‐α/MIP‐2/MMP9 signalling pathway

**FIGURE 6:**
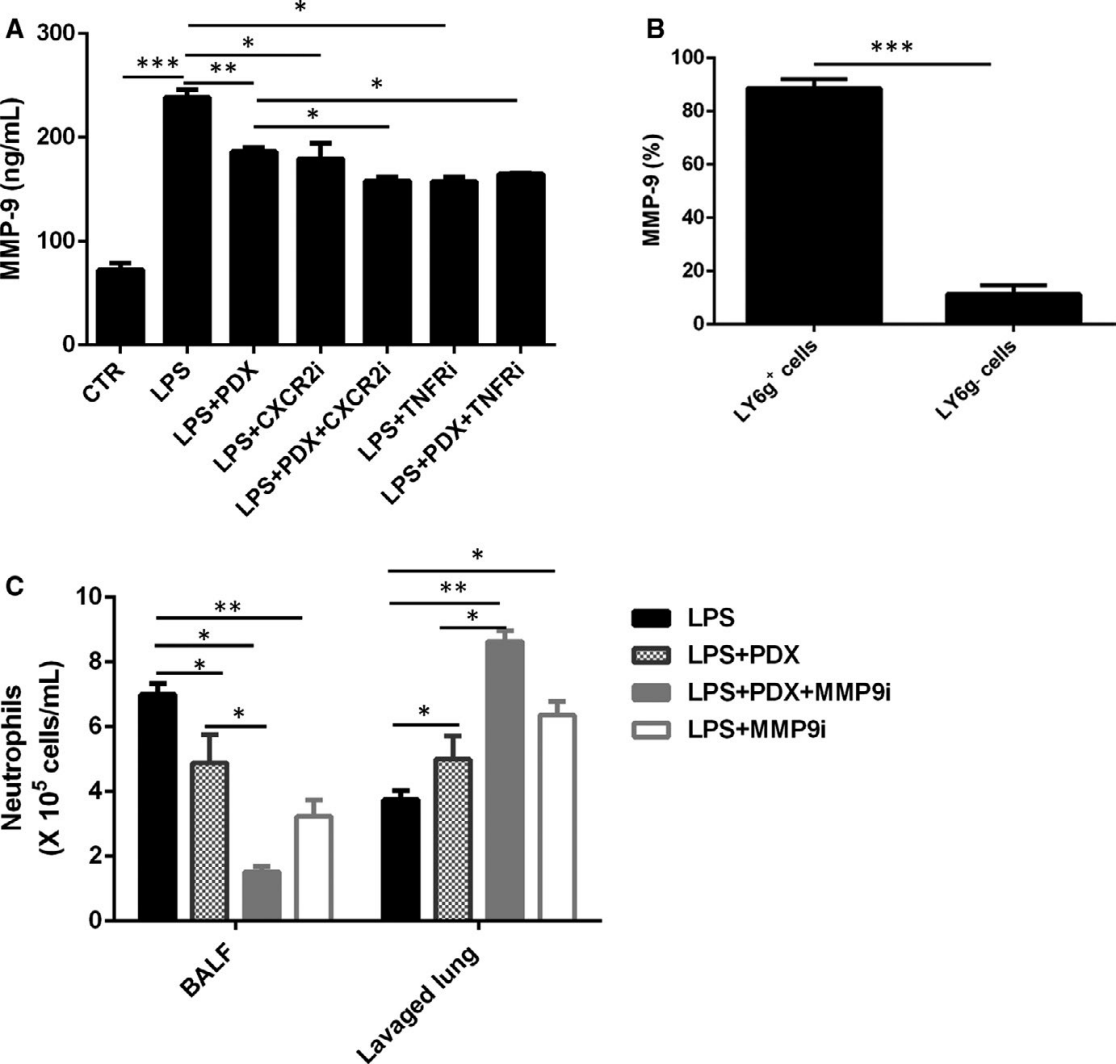
PDX regulated neutrophil transmigration into the alveolar space in connection with TNF‐α/MIP‐2/MMP9 signalling pathway. A CXCR2 inhibitor (2 mg/kg), TNFR inhibitor (15 mg/kg) or MMP9 inhibitor (15 mg/kg) in the presence or absence of PDX was administered via intraperitoneal injection after LPS administration. MMP9 expression in the BALF was measured by ELISA (A). MMP9 expression on cells was assessed by flow cytometry (B). Neutrophil numbers in the BALF and lung tissues (C) were measured by flow cytometry. Data are presented as the mean ± SEM. n = 6‐8. *P < .05, **P < .01, ***P < .001

The MMP‐9 level was reduced in the LPS + PDX group, LPS + CXCR2i group and LPS + TNFRi group compared with the LPS group (*P* < .05) (Figure 6A). Moreover, MMP‐9 level was decreased in the LPS + PDX+CXCR2i group and LPS + PDX+TNFRi group compared with the LPS + PDX group (*P* < .05) (Figure 6A). We also found that MMP‐9 was mainly produced by Ly6g^+^ cells (>90%) (Figure 6B).

In addition, the neutrophil numbers in the BALF were much lower than those in the lung homogenate in the LPS + PDX+MMP9i group (*P* < .05) (Figure 6C). Compared with those in the LPS + PDX group, the neutrophil numbers in the LPS + PDX + MMP9i group were decreased in the BALF and increased in the lung homogenate (*P* < .05) (Figure 6C).

## 4 DISCUSSION

In the present study, two LPS‐induced lung injury models were created, one established by atomization inhalation of LPS and the other established by intraperitoneal injection of LPS. We found that both approaches could significantly damage the lungs and increase lung TNF‐α expression. Moreover, 2 hours after LPS stimulation, neutrophils started infiltrating into the lungs and recruited macrophages followed at 6 hours after LPS stimulation. PDX effectively alleviated lung injury, down‐regulated TNF‐α concentration and inhibited inflammatory cells recruitment. However, the data were much more stable after LPS inhalation; therefore, the LPS inhalation‐induced lung injury model was used in our experiments.

In the present study, we showed that recruited macrophage and neutrophil numbers decreased when the resident macrophages were depleted, indicating that resident macrophages are associated with inflammatory cell accumulation. Our work was consistent with previous report that resident macrophages recruit helper macrophages into the infected bladder.^22^ PDX had no effect on inflammatory cell numbers when the resident macrophages were depleted, suggesting that PDX reducing inflammatory cell accumulation depends on resident macrophages.

MIP‐2 and MCP‐1 play crucial roles in inflammatory cell infiltration, and increased expression of MIP‐2 and MCP‐1 has been reported in various pulmonary diseases, including chronic obstructive pulmonary disease,^23^ ARDS^24^ and asthma.^25^ We have reported that MIP‐2 recruited neutrophils to the damaged lung.^26^ A previous report also demonstrated that MCP‐1 recruited inflammatory monocytes to facilitate breast tumour metastasis.^27^ Here, we showed that the expression of MIP‐2 and MCP‐1 was rapidly induced in LPS‐induced lung injury and PDX abolished the LPS‐induced up‐regulation of lung tissues MIP‐2, MCP‐1 mRNA and BALF MIP‐2, MCP‐1 protein levels, but PDX had no effect on CXCR2 and CCR2 mRNA expression, indicating that PDX reduces MIP‐2, MCP‐1 production and release, but not CXCR2 and CCR2. Moreover, the repressive effects of PDX on neutrophil numbers were abolished by CXCR2 inhibitor, and recruited macrophage numbers were abolished by CCR2 inhibitor, suggesting that PDX inhibits neutrophil accumulation via MIP‐2, and suppress recruited macrophage infiltration through MCP‐1. Since MIP‐2 and MCP‐1 were important in inflammatory cells infiltration, we asked which cells secrete MIP‐2 and MCP‐1. We found that MIP‐2 and MCP‐1 were mainly present on resident macrophage. PDX down‐regulated MIP‐2 and MCP‐1 level, indicating that PDX could reduce MIP‐2 and MCP‐1 expression on resident macrophage to repress inflammatory cell accumulation.

TNF‐α is a central mediator of inflammation and plays an important role in the host response to injury, but overexpression of TNF‐a can result in severe tissue damage and underlies a number of disease states, such as rheumatoid arthritis, ARDS and malignancy.^28^, ^29^ In the present study, we found that PDX could inhibit TNF‐α expression to protect lung tissues, which was consistent with a previous study showing that PDX abolished zymosan‐A‐induced TNF‐α production.^30^ We also found that TNF‐α was mainly expressed on recruited macrophages and worked by binding with TNFR, which was expressed on resident macrophages. Our work was consistent with previous research, which showed that chemical or genetic depletion of macrophages suggested that early recruited macrophages expressed TNF‐α^31^ and indicated that recruited exudative macrophages produced TNF‐α after stimulation with LPS.^32^ We also found that PDX inhibiting the MIP‐2 level and neutrophil numbers was associated with TNF‐α.

MMP9 derived from neutrophils during the inflammatory process can change alveolar capillary permeability and mediate neutrophil transmigration into the alveolar space.^33^ In the present study, we found that MMP9 was mainly expressed on Ly6g^+^ cells, which were identified as neutrophils. PDX reduced MMP9 expression, which was consistent with a previous study showing that MMP‐9 expression was reduced in the corneas of HSV‐1‐infected mice by treatment with AT‐PDX.^34^ Interestingly, in our study, TNFR inhibitor and CXCR2 inhibitor could inhibit neutrophil infiltration and MMP9 expression with or without PDX in LPS‐induced lung injury, indicating that MMP9 expression on neutrophils could be regulated by TNF‐α and MIP‐2. In vivo studies have shown that TNF‐a can induce the overexpression of MMP9 in cholangiocarcinoma cell lines.^35^ Moreover, an MMP9 inhibitor significantly reduced neutrophil transmigration into the alveolar space after treatment with PDX. Collectively, these data indicated that PDX mediated neutrophil transmigration into the alveolar space in connection with TNF‐α/MIP‐2/MMP9 signalling pathway.

**FIGURE 7:**
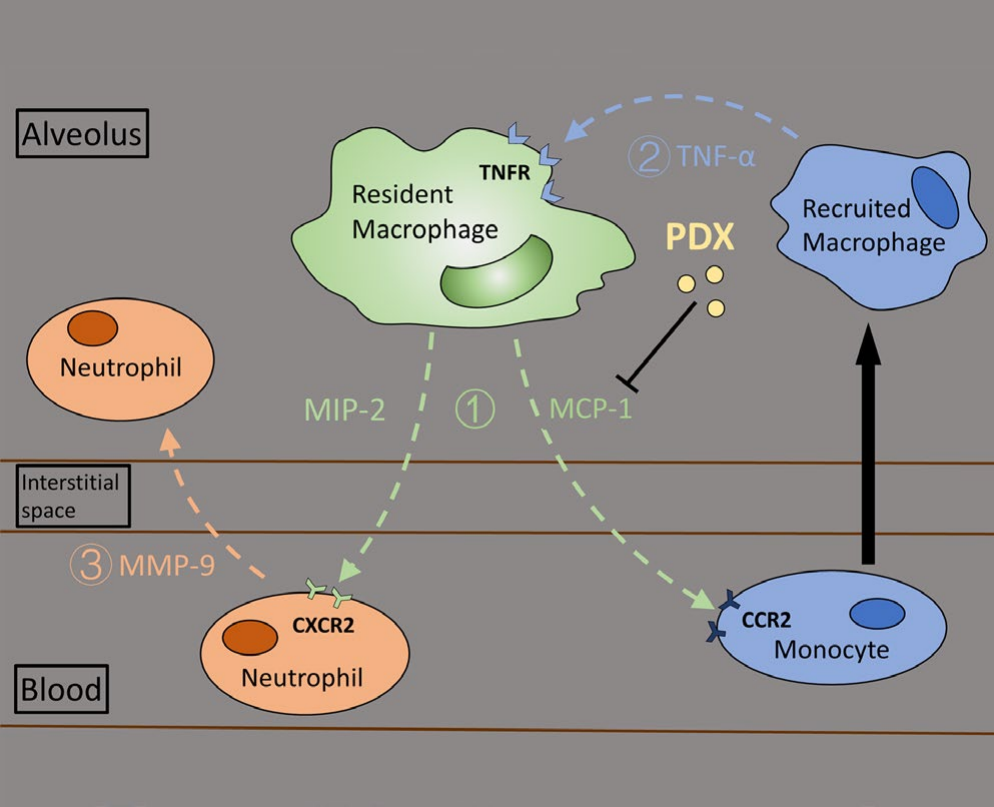
Graphical summary of the sequence of events. Our findings document the following sequence of events (Figure 7): 1) resident macrophages sensed LPS stimulation and produced the chemokine MIP‐2 and MCP‐1, which recruited neutrophils and recruited macrophages; 2) recruited macrophages produced TNF‐α; 3) TNF‐α and MIP‐2 caused MMP9 expression in neutrophils, which allowed these cells to transmigrate into the alveolar space; and 4) these processes were regulated by PDX

In conclusion, we have shown that PDX alleviates lung injury through inhibition of MIP‐2 and MCP‐1 production and release by resident macrophages in LPS‐induced lung injury. PDX inhibited recruited macrophage TNF‐α expression. We also found that PDX inhibited neutrophil infiltration in connection with TNF‐α/MIP‐2/MMP9 signalling pathway (Figure 7). Our findings suggest that PDX may provide a new therapy for the treatment of ARDS.

## CONFLICT OF INTEREST

The authors confirm that there are no conflicts of interest.

## AUTHOR CONTRIBUTION

**Yang Ye:** Investigation (equal); Methodology (equal). **Hua‐Wei Zhang:** Investigation (equal); Methodology (equal). **Hong‐Xia Mei:** Data curation (lead). **Hao‐Ran Xu:** Data curation (lead). **Shu‐Yang Xiang:** Data curation (supporting). **Qian Yang:** Writing‐original draft (lead). **Sheng‐Xing Zheng:** Writing‐original draft (supporting). **Fang Gao Smith:** Writing‐original draft (lead). **Sheng‐Wei Jin:** Conceptualization (equal); Writing‐review & editing (equal). **Qian Wang:** Conceptualization (equal); Writing‐review & editing (equal). 

## Data Availability

I confirm that my article contains a Data Availability Statement even if no data are available (list of sample statements) unless my article type does not require one (eg Editorials, Corrections, Book Reviews, etc). I confirm that I have included a citation for available data in my references section, unless my article type is exempt.
